
JOURNAL INFORMATION
==============================
NLM Title Abbreviation: Wirtschaftsdienst
Journal ID: Wirtschaftsdienst
NLM Journal ID: 101086612

Wirtschaftsdienst (Hamburg, Germany : 1949)

ISSN: 0043-6275

ARTICLE INFORMATION
==============================
PMCID: PMC7813611
PMID: 33487765
DOI: 10.1007/s10273-021-2812-x
Article ID: 2812
Article version: 1
Subjects: Kurz Kommentiert

Ökonomik der Pandemiepolitik

Kollektivgut

Collective good: the economics of pandemic policy

Funk Lothar lothar.funk@hs-duesseldorf.de

grid.440973.d 0000 0001 0729 0889 Fachbereich Wirtschaftswissenschaften, Hochschule Düsseldorf, Münsterstraße 156, 40476 Düsseldorf, Deutschland
Electronic publication date: 2021 Jan 19
Print publication date: 2021
Volume: 101
Issue: 1
First page: 6
Last page: 6
Copyright: © Der/die Autor(en) 2021
License: Open Access: Dieser Artikel wird unter der Creative Commons Namensnennung 4.0 International Lizenz (https://creativecommons.org/licenses/by/4.0/deed.de) veröffentlicht.
Open Access wird durch die ZBW — Leibniz-Informationszentrum Wirtschaft gefördert.
License URL: https://creativecommons.org/licenses/by/4.0/

issue-copyright-statement: © The Author(s) 2021

==============================
DOI: 10.1007/s10273-021-2812-x

Kollektivgut

### Ökonomik der Pandemiepolitik

Freie private Märkte stellen Produkte mit Kollektivguteigenschaften wegen Problemen des Trittbrettfahrerverhaltens von eigennutzorientiert rational handelnden Personen oft nicht optimal bereit. Es gibt nicht nur reine Kollektivgüter mit Geltung von zugleich Nichtrivalität und Nichtausschluss beim Konsum, sondern auch partielle. Bei Letzteren ist nur je eines dieser Kriterien erfüllt: Allmendegut (kein rationierter Zugang, Konsumrivalität) und Mautgut (rationierter Zugang; keine Konsumrivalität). Durch diese Brille betrachtet, handelt es sich bei dem Kollektivgut „individuelle Freiheit" – z.B. in der Fußgängerzone zu üblichen Geschäftszeiten einzukaufen oder ein Restaurant zu besuchen – um ein reines Kollektivgut. Von der privaten Nutzung solcher Freiheiten kann in Deutschland niemand staatlich ausgeschlossen werden, und es gibt dabei normalerweise auch keine Probleme beim Zugang (Nichtrivalität). Durch Corona ist aus diesem grundsätzlich öffentlich verfügbaren Raum jedoch aus ökonomischer Sicht ein Allmendegut geworden, bei dem der Staat zur Wahrung des öffentlichen Gesundheitsschutzes massiv regulierend eingegriffen hat. Denn eine Steuerung über individuell-verantwortliches freiwilliges Verhalten und politische Appelle erwies sich als unzureichend.

Dass strengere Eingriffe erforderlich sind, war aus der verhaltensökonomischen Empirie für die kältere Jahreszeit klar vorhersehbar, wenn staatlich gesetzte maximale Infektionsziele bei der Verlagerung des Lebens in geschlossene Räume mit erhöhter Infektionsgefahr auch, trotz technischer Vorkehrungen zur Ansteckungsvermeidung, tatsächlich erreicht werden sollten. Die nötigen Sanktionen bei Regelverstößen, etwa Bußgelder bei Missachtung eines adäquaten Mund-Nasen-Schutzes oder verordneter Quarantäne, erschienen zunächst angesichts des von Opposition, Lobbyisten und juristisch oft angeführten Maßstabs der Verhältnismäßigkeit für Regierende politisch schwer durchsetzbar. Insgesamt wurde die vorherige staatliche (Teil-)Kompensation der unverschuldeten Verlierer dieser Krise zunehmend ergänzt durch staatliche Sanktionen, um so einen ausgewogeneren Mix aus Zuckerbrot und Peitsche zu ermöglichen.

Die Empirie zu mangelnder Rücksichtnahme bei der Nutzung von Kollektivgütern generell und speziell zur Einhaltung von Hygieneregeln in Corona-Zeiten verdeutlicht: Trittbrettfahren ist zwar nicht so verbreitet, wie es das spieltheoretische Gefangenendilemma prognostiziert. Dennoch neigen bis zu 30% der Bevölkerung zu „Free Riding". Ein Mangel an wirksamen Maßnahmen zu dessen Eindämmung verschlimmert unzureichende Regeltreue sogar. Ein Teil der Menschen, die ansonsten Regeln überwiegend einhalten, verhält sich bei ständig folgenlosen Zuwiderhandlungen anderer ebenfalls zunehmend regelinkonform. Das Allmendegut in der Corona-Krise, bei dem der Staat regelnd eingreifen kann, ist in erster Linie der öffentliche Raum, der jedem Einzelnen zur Verfügung steht. Gerade durch massive Verstöße gegen die Corona-Regeln im öffentlichen Bereich, etwa bei Demonstrationen gegen Regierungsmaßnahmen (und der An- und Abreise), kann es zu negativen externen Effekten kommen, etwa indem noch symptomlose Infizierte andere anstecken. Menschen, die sich selber an die Regeln halten, können dabei angesteckt werden. Womöglich können sie nicht sofort behandelt werden, weil es zu wenig Kapazitäten in Krankenhäusern gibt. Letztere sind folglich auch Allmendegüter, durch deren Nutzung Rivalität zwischen verschiedenen potenziellen Nutzern (schwere Verläufe bei COVID-19 oder auch andere akut schwere Erkrankungen) entsteht, jedoch ein Ausschluss eines Nutzungsberechtigten nicht möglich ist. Regelverstöße sind natürlich auch bei privaten Festen mit Familie und Freunden möglich, bei denen die nötige Vorsicht oft schwerfällt und eine Regeleinhaltung kaum kontrollierbar ist.

Der Gutscharakter lässt sich in ein Mautgut ändern, indem über das Ausschlussprinzip rationiert wird. Dies ist durch die Verhängung von Bußgeldern etwa bei Verstößen gegen die Maskenpflicht im öffentlichen Raum bereits geschehen. Dies kann ein verhältnismäßiges Rationierungsinstrument sein, um Trittbrettfahren zu vermeiden. Denn hier kommt es zu relativ geringen individuellen Freiheitsbeschränkungen, verglichen mit den so gewonnenen Spielräumen für andere Freiheiten. Denkbar wäre es auch, dass der Staat eine Überforderung des öffentlichen Raums durch zu viel potenziell ansteckende physische Nähe infolge von Personenanhäufungen dadurch unterbindet, dass intelligente Lenkungsmechanismen zu einer Entzerrung des Gedränges im öffentlichen Nahverkehr, an Schulen, beim Einkauf (etwa auch über spezielle Öffnungszeiten für Ältere und andere Risikogruppen) eingesetzt werden, um Infektionen zu vermeiden. Dies könnte effizienter sein als sehr weitgehende administrative Schließungsgebote und Öffnungsverbote. Angesichts des auch globalen Kollektivgutcharakters von Corona sind Mutationen mit schnellerer Ausbreitung alarmierend. Denn laut der Standardökonomik verfügen in dieser Lage „weder die Marktteilnehmer noch die nationalen Regierungen über angemessene Anreize, um ein effizientes Ergebnis zu erzielen", wie es im VWL-Klassiker von Samuelson/Nordhaus heißt.

Lothar Funk
Hochschule Düsseldorf
lothar.funk@hs-duesseldorf.de

© Der/die Autor:in(nen) 2021. Open Access: Dieser Artikel wird unter der Creative Commons Namensnennung 4.0 International Lizenz (https://creativecommons.org/licenses/by/4.0/deed.de) veröffentlicht.

Open Access wird durch die ZBW – Leibniz-Informationszentrum Wirtschaft gefördert.
